# “Feeling clean”: stigma and intravaginal practices among female entertainment workers in Cambodia

**DOI:** 10.1186/s12905-021-01271-y

**Published:** 2021-03-25

**Authors:** Carinne Brody, Rachel L. Berkowitz, Pheak Chhoun, Kathryn C. Kaplan, Sovannary Tuot, Siyan Yi

**Affiliations:** 1 Public Health Program, College of Education and Health Sciences, Touro University California, Vallejo, CA 94592 USA; 2 School of Public Health, University of California, Berkeley, CA USA; 3 KHANA Center for Population Health Research, Phnom Penh, Cambodia; 4 Saw Swee Hock School of Public Health, National University of Singapore and National University Health System, Singapore, Singapore

**Keywords:** Sex work, Intravaginal practices, Cambodia, Douching, Stigma

## Abstract

**Background:**

Intravaginal practices (IVPs), methods used by women most often to manage vaginal hygiene and address perceived disruptions to vaginal health, may increase the risk of contracting human immunodeficiency virus (HIV) and other sexually transmitted infections (STIs). This qualitative study explores the social, professional, and peer context surrounding IVPs, the experiences of self-cleaning or getting cleaned from a health professional, and the perceived impacts of IVPs among female entertainment workers (FEWs) in Cambodia.

**Methods:**

In 2017, we conducted 27 focus group discussions from four provinces, and 16 follow-up semi-structured in-depth interviews with purposively selected participants in two provinces. Data collection occurred over three weeks, with concurrent data transcription and translation. The data from the transcripts were analyzed using Dedoose, an online, open-access qualitative analysis software. Two researchers independently labeled sections of transcripts associated with broader categories and subcategories based on the initial content analysis matrix and created codes. This process continued iteratively until a final coding schema and conceptual model was created.

**Results:**

We found that IVPs are widely practiced among FEWs in Cambodia and are associated with internalized and enacted stigma. Stigma was an overarching theme that impacted the sub-themes of (1) messages about cleaning, (2) the cleaning process, and (3) the impact of cleaning. Experiences of enacted stigma and internalized stigma permeated conversations about IVP, including feeling pressured by peers to keep themselves clean, practicing internal cleaning after transactional sex, and being called dirty by health providers.

**Conclusions:**

FEWs who practice IVP talk about it in the context of their lived experiences stigma and discrimination. Highly stigmatized practices such as IVP among FEWs may benefit from a harm reduction approach that emphasizes positive changes without judgment, coercion, or discrimination.

## Background

Intravaginal practices (IVPs) refer to methods used by women, frequently to manage vaginal hygiene and address perceived disruptions to vaginal health [1, 2]. IVPs consist of intravaginal washing or douching with liquids such as water, water with soap, or household cleaning products, wiping inside the vagina with cloth or tissue, and applying or inserting substances with the intent to warm, dry, or tighten the vagina [3–5]. Evidence suggests a wide range of IVPs among the general public across the globe [6]. However, the prevalence of IVPs is higher among female entertainment workers (FEWs), women who work at entertainment venues where they sing, dance, flirt, give massages and sometimes engage in transactional sex. [1, 3, 7–9].

Women use IVPs for perceived hygienic and health-promoting reasons despite IVPs having been linked to adverse health effects [1]. IVPs may contribute to disease via several mechanisms: they may disrupt the genital mucosa and cause vaginal and cervical inflammation, they may disrupt normal vaginal flora and allow the growth of pathogens, or they may provide a vehicle for the transport of a pathogen allowing lower genital tract infections to spread beyond the cervix and into the uterus, fallopian tubes, or abdominal cavity [1, 10–12]. As a result, IVPs can increase the risk of contracting sexually transmitted infections (STIs) and other vaginal infections, which may increase the risk of human immunodeficiency virus (HIV) transmission [1, 4, 12]. In addition, IVPs have been implicated in numerous adverse reproductive health outcomes, including increased risk of pelvic inflammatory disease, reduced fertility, ectopic pregnancy, preterm delivery, low birth weight, and cervical carcinoma [13–15].

Studies in Asia, Africa, and the Americas reveal that utilization of IVPs is widely prevalent within the sex and entertainment industry. These practices are utilized by anywhere from 40% to sometimes nearly 100% of FEWs as well as sex workers, depending on the region [3–9, 16–18]. In Cambodia, studies have shown that IVPs are used by 77% of females in the general population and up to 91% of FEWs [3, 19]. One study in Cambodia showed that FEWs do not only more frequently practice IVPs, but they use IVPs that are more diverse in type and method [16]. Additionally, as FEWs are more likely to be exposed to STIs and to develop vaginal infections than the general public, they are exceedingly more susceptible to HIV infection and adverse reproductive health outcomes [3].

FEWs stated several motivations for using IVPs. The motivations include wanting to prevent STIs, promoting cleanliness/avoiding odors, relieving/treating symptoms of illness, promoting general health and well-being, freshening/tightening their vagina to increase sexual pleasure for their clients, and preventing unwanted pregnancies [2, 3, 17, 18, 20–24]. Some studies found that FEWs perceive IVPs as their duty as women or as sex workers to be presentable for their clients [3, 24]. Most women reported utilizing IVPs once they entered into sex work or within one or two years of beginning sex work. FEWs reported performing IVPs before and after having sex with clients [3]. Frequencies of IVPs among FEWs can be from one to 28 times per week [3, 18, 20]. FEWs have reported learning about IVPs from their mothers [7], friends, and healthcare providers [3]. Beyond self-cleaning practices, FEWs in Cambodia have reported getting professional IVP services offered in clinics or salons [3, 20].

This qualitative study explores the social, professional, and peer context surrounding IVPs, the experiences of self-cleaning or receiving services from a health professional, and the perceived effects of IVPs among FEWs in Cambodia.

## Methods

This analysis was part of the formative study for the development of the Mobile Link, a randomized controlled trial aiming to improve sexual and reproductive health of FEWs in Cambodia. The trial aims to engage FEWs through messages on their mobile phones and link them to the existing high-quality prevention, care, and treatment services in the country. We have published the details of the Mobile Link project’s protocol elsewhere [25]. In short, the research team gathered information on knowledge, attitudes, and practices related to sexual and reproductive health among FEWs in the four provinces with the highest HIV burden in Cambodia to inform the Mobile Link message creation and program implementation [26]. For this study, we examined transcripts of the focus group discussions (FGDs) and in-depth interviews (IDIs), where women discussed IVPs.

Researchers at a Cambodian health organization, KHANA, who have been working with FEWs for over 10 years, led this project. FGD and IDI guides were co-developed by the research team with the program team and peer outreach workers. Questions were open-ended, and the topics included in the guides covered a wide range of sexual and reproductive health topics, including HIV, gender-based violence, and substance use. We conducted a two-day training for the two female data collectors, a peer data collector and a community lay health worker, in each province. The training provided guidance on the objectives of the overall project including how to use the FGD and IDI guide tools and basics of conducting FGDs and IDIs, especially with vulnerable populations.

The guides were also piloted in each province and revised based on local feedback. We trained the moderators to follow the guides and gave them the authority to use facilitation techniques to encourage participant interaction even if it went off-topic. Field notes were taken and used to incorporate changes into the final versions of the tool following the project team debriefing after each pilot FGD.

### Sampling and site selection

In each city, we used a two-stage cluster design to identify entertainment venues. First, we randomly selected a venue from a list of entertainment venues in the province by venue type (karaoke bars, massage parlors, beer gardens, bars, restaurants). Then, outreach workers recruited FEWs to participate by approaching them at their place of work before work hours. Eligibility requirements for participation were the following: working in entertainment in Cambodia, between the ages of 18 and 30, currently sexually active, in possession of a mobile phone and can send/receive/respond to a text message and able to give their informed consent. Once a participant agreed to participate, her contact information was collected and she was given the time and place to meet for the focus group or interview.

### Data collection process

#### Focus groups

Focus group data collection occurred in four provinces over three weeks, with concurrent data transcription and translation. The project coordinator oversaw the data collection activities. Before arrival at the organization’s community center where the data collection took place, the data collector worked with the peer data collector to organize logistics. The community health worker contacted all recruited participants to ensure they would arrive on the day of the data collection. Upon arrival, participants were permitted to view and casually discuss topic listed on cards around the room hung by the community health worker. The data collector read the informed consent to the participants, asked for their verbal consent, signed as witnesses and gave a copy of the consent form. No one else was present during the data collection aside from the two data collectors and the participants.

Each FGD lasted around 90 min and covered various topics. The topics included STIs, HIV, modern contraception, gynecological health, condom use, cancer, pregnancy, gender-based violence, and substance use. Within each topical area, questions explored participants’ understanding of the topic area, known myths/misconceptions, practices related to the topic and how the health information and linkages could be used to improve outcomes related to that issue. Women were encouraged to share specific experiences such as stories from their own lives to generate conversation. Details about the methodology of the focus groups including the focus group guide have been previously published [27]. Field notes were taken during the focus group discussions to capture non-verbal cues as well as track themes and patterns in responses. Revision workshops were conducted where researchers provided a summary of the data collected during the FGDs to the participants in order to get their feedback and confirmation of results.

#### Follow-up interviews

After the initial FGD transcripts were analyzed, the research team decided to go back to the study population to learn more about intravaginal practices as this was identified during the FGDs as a top health issue. The data collection team then conducted semi-structured IDIs with participants who were available at randomly selected venues in Phnom Penh and Siem Reap during pre-work hours. An interview guide was developed and pilot tested based on the IVP themes from the focus groups and workshops. Questions were asked about where participants have received advice about cleaning, motivations to clean, self-cleaning practices, professional cleaning services and frequency of cleaning. The full interview guide developed for the follow-up interviews is provided as Additional File 1. Data collectors continued to interview participants until they felt they had reached data saturation.

All FGD, workshop and IDs were audio recorded and audio files were uploaded into a locked Dropbox managed by the project coordinator. Transcribers accessed the audio files, transcribed the data into Khmer, and uploaded Khmer transcripts into a second Dropbox folder. Translators then accessed the Khmer transcripts to translate into English and upload the English file into the final Dropbox folder. A total of 27 FGD with 8 participants in each group and 16 IDI transcripts formed the data set for this project for a total of 232 participants. Approximately 10% of those approached declined to participate in the study due to being busy at work and not having enough time to join.

#### Transcription and translation

All audio files were transferred into a password-protected digital folder by the project coordinator. Transcribers were granted access to the audio files where they transcribed the data into Khmer language. Then the Khmer transcripts were uploaded into a second digital folder. A team of translators read the Khmer transcripts and translated them into English. The final English transcripts were read by the project coordinator for clarity and any clarity issues were dealt with by referring to the original audio file.

### Data analyses

The data from the transcripts were analyzed using Dedoose Version 8.0. 35, a web application for managing, analyzing, and presenting qualitative and mixed method research data (2018). The analysis followed thematic analysis procedures, and two researchers developed codes inductively based on participant comments, which addressed the research questions [28]. Secondly, we used thematic coding to identify themes that illuminated the influences, experiences, and cleaning processes from the participants’ perspectives. The first two authors developed the codebook iteratively based on the first round of coding of a sample of FGDs and IDIs and then coded the rest of the transcripts. Then, the codebook was applied to the 16 follow-up IDIs once those were completed. The codebook was refined and amended to encompass any new information gleaned from the follow-up interviews. Finally, a conceptual model was developed based on the codebook which depicts the authors impression of how the codes all relate to each other.

## Ethical consideration

This study was approved by the National Ethics Committee for Health Research (NECHR, No. 142NECHR) within the Ministry of Health in Cambodia and the Touro College Institutional Review Board (No. PH-0117). All members of our research team have undergone a research ethics training on the protection of human research participants through the National Institute of Health (NIH) online training program. All FGD and IDI participants went through an informed consent process where they were told the study purpose, the risks and benefits to their participation and the voluntary nature of their participation. The data collector read the informed consent to the participants, asked for their verbal consent, signed as witnesses and gave a copy of the consent form. Each participant was given copies of the consent form, translated into Cambodia language (Khmer). The data collectors ensured that the FGD and IDI spaces were private and confidential. This study required some participants to discuss sensitive topics in a group setting. Participants were informed of the special issues of confidentiality that come with the focus group study design. In addition, participants were offered escorted referrals to counselors and health services including to a local women’s center.

## Results

**Fig. 1:**
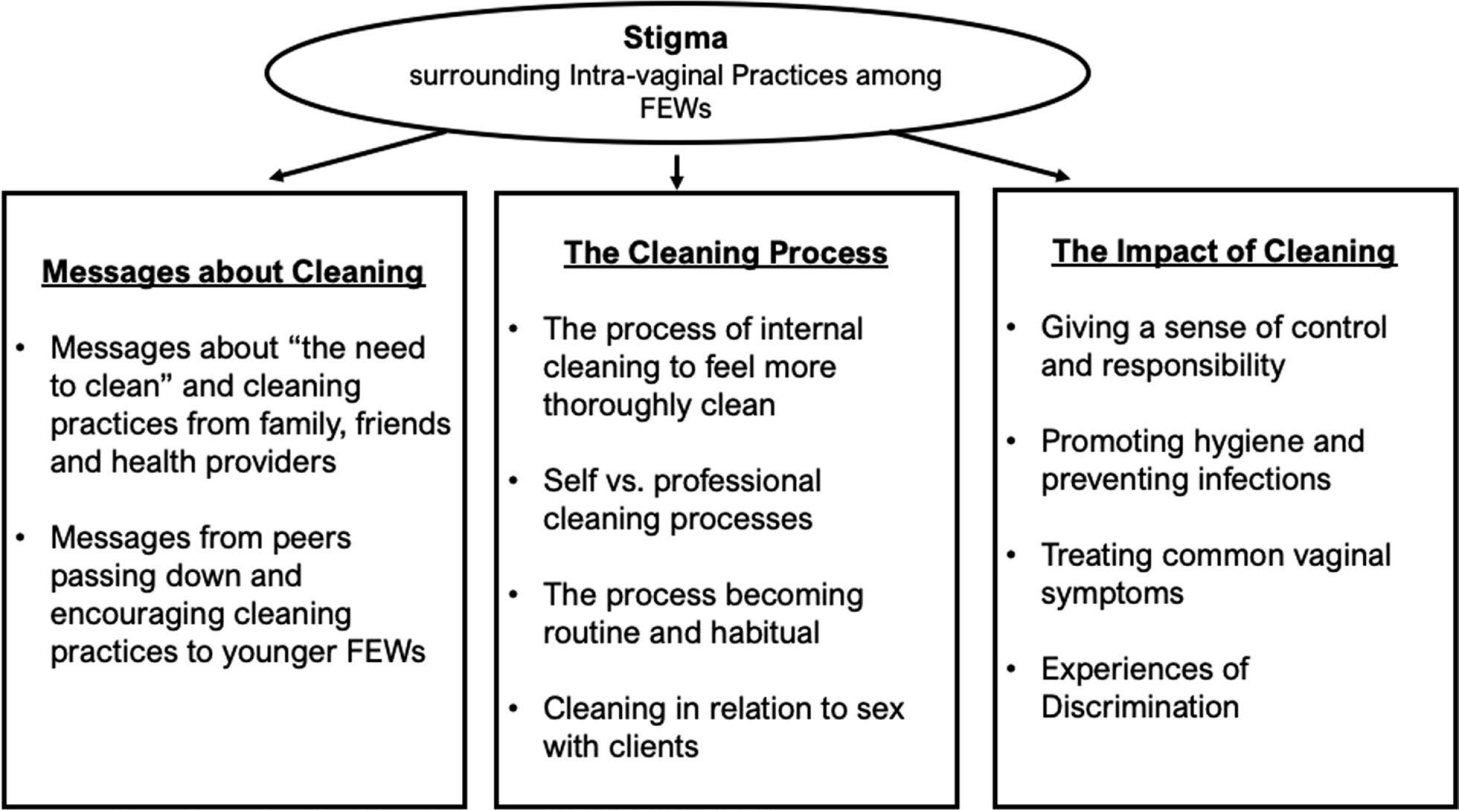
Conceptual model for themes around intravaginal practices

Figure 1 depicts the relationships between the main themes and sub-themes. Stigma is an overarching theme that impacts messages about cleaning, the cleaning process, and the impact of cleaning.

### Stigma in intravaginal practices

Stigma was a persistent thread throughout this analysis. Experiences of enacted stigma as well as internalized stigma permeated conversations about intravaginal practices.

Experiences of enacted stigma (discrimination) were linked to cleaning, such as being told that FEWs are dirty by health providers.

The doctor from the clinic and his colleague said that we have dirty, stinky vaginas, and we don’t come and get them cleaned, without us leaving completely. (Phnom Penh, FGD)

Internalized stigma was also apparent when women talked about the causes of gynecological issues among FEWs. Women talked about their own colleagues ignorance, laziness, dirtiness, and willful refusal to practice IVP as primary causes rather than their limited health care access, socioeconomic status or work environments.

Respondent 1: Some FEWs just don’t know how to protect and clean.

Respondent 2: Sometimes, after they have sex with clients, they don’t clean their vagina and keep it all day. (Siem Reap, FGD)

### Messages about IVPs

Women described a landscape of many sources of varying information about whether and how to wash the vagina and the potential benefits and consequences of washing. The information was often contradictory. Elders, family, peers, doctors were all mentioned as sources of information and advices about cleaning.

### From family and community

Several women shared stories of learning about washing from members of their community, information that was passed down to them about how to care for their bodies.

Moderator (M): How long have you known about that salt and lemon technique?

R: A long time ago. Elder people talk about it, the elder Khmer people told me (Phnom Penh, IDI).

### From other FEWs

FEWs spoke about sharing cleaning practices almost as if they were passing down such knowledge is a part of their professional duties.

My sister always wants the doctor to check on her, and do the cleaning. She said FEWs are supposed to do the [health] checkups of the uterus every six months, depending on our [availability]. Therefore, I follow her routine, as well.” (Phnom Penh, IDI)

Then we explain it to the newcomers since they don’t know. (Battambang, FGD)

### From health providers

Women shared practices and advice received from health providers about cleaning. Women reported that doctors both encouraged and discouraged cleaning practices. Women stated that doctors suggested that they clean to maintain hygiene without being detailed about how, or in some cases, encouraged them to seek professional services for cleaning.

In the excerpts below, women shared information they received from doctors discouraging them from washing:

I was told that too much cleansing can cause leucorrhea so the doctor told us to use normal water instead since cleansing gel may cause irritation if not properly cleaned. (Siem Reap, FGD)

In contrast, other women reported receiving advice about how important self-cleaning is to maintaining hygiene:

[The doctor] told me that as a woman, I have to know how to keep proper hygiene and protect myself at home. So, I clean myself frequently. (Phnom Penh, IDI)

While the above example shows how some doctors might express support for some forms of at-home cleaning, the example below underscores the lack of clarity about what washing entails:

M: And where do you get that cleaning technique from?

R: I went to the hospital, and the doctor told me to clean and protect myself from infection, something like that.

The excerpts above allude to advice about at-home cleaning as a part of maintaining hygiene. However, in the excerpts below, women discussed how doctors advised them regarding professional cleanings:

Before I went [for cleaning services], I felt afraid because I had never done that before. I had never had a cleaning like that before. But when I arrived there, the doctor explained to me this and that and told me not to worry, because it is normal, and for the gynecological disease, it requires some care of the vagina to be cleaned. For us, women, it is very important. (Siem Reap, IDI)

Another woman echoed this idea, describing an interaction where she was told that a professional cleaning would prevent STIs and maintain her hygiene:

“They said it would prevent me from getting an STI, and it would keep my hygiene good.” (Battambang, IDI)

Overall, FEWs navigate a complex landscape of messaging about whether and how to clean. Cleaning may be a norm shared by their communities or family members. It might also be a practice actively encouraged and supported by peers in the same profession. Those who do not use such practices may be frowned upon or judged by peers. In addition, health care providers responsible for providing gynecological care seem to offer somewhat mixed guidance. FEWs report receiving mixed information from health care providers. They are hearing that some aspects of IVP are beneficial while other aspects are not. Health care providers also appear to be offering professional cleaning services and discouraging at-home cleaning.

## Cleaning process

In the context of this complicated landscape, women shared the practices they used to clean. Women used a diverse set of approaches and materials used to support cleaning. They identified two modes of cleaning: self-cleaning, or the use of professional services. These practices, as described by participants, are detailed below.

### Self-cleaning process

Self-cleaning is the practice of inserting materials into one’s own vagina with the intention of cleaning the vagina and, for some women, the cervix or uterus. As illustrated by the quotes below, from interviews and FGDs, women’s self-cleaning practices and the types of materials used vary:

We clean with tamarind water, or saltwater, a little bit warm, and use a piece of soft cloth, and use my hand to scrub. And at the end, I use the fresh warm water to clean it. (Banteay Meanchey, IDI)

### Professional cleaning process

Professional cleaning involves a doctor or other provider in a healthcare setting such as a hospital or clinic inserting materials (e.g. tweezers, cloth, cotton, and some kinds of liquid) into a woman’s vagina for the purpose of cleaning the vagina, cervix, and/or uterus. Women shared what happens during those sessions:

R: They told me to go lay down on the bed, and then they scrubbed with water, then they cleaned the inside for me.

M: When they cleaned inside for you, what did they use to clean inside for you?

R: The tweezer, and they wore gloves. They cleaned in a circle. They used cloth (Phnom Penh, IDI)

The experience of being cleaned was described by some women as mildly painful or unpleasant, while others described feeling fresh and clean as a result of the cleaning.

I changed my clothes. Then the doctor started cleaning my uterus. I felt very fresh and clean, unlike before I went there. (Battambang FGD)

While some women exclusively practiced self-cleaning or professional services, other women indicated a cyclical relationship between self-cleaning and professional services.

R1: Some women clean only the outside, but then the inside is still not clean.

R2: We can clean only the outside. So we want to get cleaned on the inside at the hospital once a week. (Battambang FGD)

### Frequency of cleaning

Some women described self-cleaning as part of their daily or weekly routines and professional cleaning at regular intervals (e.g., monthly or every 5–6 months). Others described cleaning as something they did before and/or after sex with partners or clients.

We washed immediately [=after sex to flush out the semen. After 30mn or 1h, male gamete would not come out (Banteay Meanchey, FGD)

## Impact of cleaning

Women had many ideas about the impact of cleaning. The ideas included the promotion of hygiene, the prevention of infection, the treatment of existing issues, and the experiences of stigma and discrimination when getting professional cleaning services. Cleaning also gave FEWS to feel a sense of responsibility, confidence, and control.

### Promotion of hygiene and prevention of infection

Women described how they used cleaning as a part of a routine to maintain health and hygiene. Health and hygiene was the most commonly cited reason for cleaning. Some women described routines as a general practice, while others connected the routines to the specific context of their work.

### Prevention of vaginal health issues

Both self-cleaning and professional cleaning were identified as necessary to prevent vaginal health issues like infections, discharge, inflammation, STIs, and fungus.

In the quotes below, women described how they used cleaning to prevent irritation or discharge that may result from sex work:

Because we have lots of clients, that’s why we need to clean. If we did not clean, we would have discharge or irritation in severe cases. (Siem Reap, FGD)

Other women thought that washing could prevent STIs:

Sometimes condoms break, but then I washed immediately. I want to know if I washed immediately right after having unprotected sex, does it prevent HIV transmission? (Siem Reap, FGD)

### Treatment of an existing issue

Both at-home and professional cleaning were commonly discussed for the treatment of vaginal health problems. Both were seen as effective at treating inflammation, STIs, discharge, itching, smell, and leucorrhea.

When I had it [referring to itchiness], I mixed salt, lemon, and water. Then I washed my genital area with it...after washing with it, I did not have itchiness anymore. (Battambang, FGD)

### Sense of confidence

Women who reported cleaning in the absence of any physical symptoms said they did so because they wanted to feel confident, clean, and fresh. Women’s descriptions of IVPs as a “good thing to do” or as something they “just want to do for themselves” suggest a sense of ownership and control over their participation. Self-cleaning was perceived by some women as an exercise in self-sufficiency, enabling them to practice self-care without needing to rely on medical resources. The ability to take care of one’s vaginal health without having to put one’s faith in others was presented as necessary.

R: We felt cleaned and confident after we got the cleaning service. (Phnom Penh, IDI)

### Experiences of stigma and discrimination

Women described how, when they went for professional cleaning, they, or their friends, were treated poorly by providers. The women below described the various ways providers may stigmatize them:

Sometimes, the doctor said he did not want to help them since we were too dirty. (Phnom Penh, FGD)

R1: They [referring to doctors] did not care when we hurt since they knew we came from the organization and so we must be the FEWs.

R2: Some doctors said that without proper washing first, they did not want to clean us. (Phnom Penh, FGD)

When I got washed at the hospital, the doctor used such bad words to me and used lots of force and hurt me. (Battambang, FGD)

Descriptions of acts where doctor’s call them dirty, deny them services until they were considered clean enough to receive care, blame them for their health problems, or are unnecessarily rough in providing services were present in most FGDs.

## Discussion

This study describes the complicated roles of intravaginal practices in the lives of FEWs in Cambodia. The themes of internalized and enacted stigma emerged as a motivation to clean, a source of pressure from peers to practice internal cleaning, and a consequence of seeking cleaning services by health providers. In summary, FEWs described a complex landscape of motivations and behaviors around IVPs. This included whether to clean, how to clean, and when to clean. If they choose not to clean, they fear repercussions about how others perceive them in their communities. If they choose to clean, they may be harming themselves or experience stigmatizing care by a health professional.

As in our study, other studies from around the world have found that discrimination and disrespect from health care providers towards entertainment and sex workers is a significant barrier to healthcare access, especially for HIV/STI testing and treatment [28–32]. Fewer studies have found internalized stigma and peer pressure as motivations for personal behaviors such as IVPs, as we found. Nevertheless, one study from China found that vaginal “cleansing rituals” were an essential part of “maintaining a clean image” among sex workers. They also noted that one who does not practice these daily cleanings was “condemned for the consequences of her behavior” [33].

Our study found that women reported cleaning themselves frequently, and this was directly related to sex with clients. A cross-sectional study among Cambodian FEWs in 2015 found that douching, which typically indicates washing the outside of the vagina, was practiced among 91% of respondents usually right after sex, especially among those who engaged in transactional sex [34]. Another qualitative study found a clear and close connection between IVPs and sex work among the same population [3].

In our study, FEWs discussed one motivation for IVPs being the prevention or treatment of infections, particularly STIs. Much of the medical literature around IVPs suggests that these practices are not associated with the prevention or treatment of infections [1]. A study on the effectiveness of an IVP cessation intervention among women living with HIV in Kenya found a decrease in vaginal candidiasis after three months of the cessation of IVP [22]. A cross-sectional study among 200 FEWs in Cambodia found that intravaginal washing in the past three months was not associated with lower HPV genotypes collected through self-collected specimens [35]. Further investigation of the role of IVPs in disease transmission is warranted.

Our study suggests that local health providers are giving mixed messages and, more importantly, delivering discriminatory care to FEWs. Women talked about providers telling them both that they do not need to practice IVP while also providing them cleaning services. This suggests that providers may need more support in delivering messaging that is clear and informative. It is possible that providers are saying they are providing cleaning services, while in reality providing other services such as checking for STIs. This area needs more investigation.

The one other published qualitative study which details IVPs among FEWs in Cambodia suggests that health providers may be “agents to change IVPs and an effective channel to deliver interventions” [3]. Our findings suggest that health providers were often discriminatory towards FEWs and promoted mixed messages about IVPs. We anticipate that much work would need to be done with providers before they can be true advocates for FEWs and deliver healthy and respectful messages around IVPs.

Interpretation of study findings are limited by some important factors including our sampling strategy and our data collection tools. We recruited participants using purposive sampling and therefore, our findings are not generalizable to the entire population of FEWs. Those who participated may have been more connected to ongoing health and outreach programs and therefore might have more information about IVP and cleaning practices. As such, our findings may under represent the lived experiences of FEWs with less information about IVP. In addition, our data collection tools (focus group and interview guides) were not validated tools because we could not find existing tools that were relevant. As a result, our questions may be leading or biased in some way that we did not intend. In response, we note that participants did not appear to be uncomfortable answering questions about IVP and conversation flowed smoothly.

## Conclusions

Our findings suggest that IVPs are widely practiced among FEWS in Cambodia and associated with internalized and enacted stigma. Highly stigmatized practices may benefit from a harm reduction approach—a strategy that aims to reduce harm associated with a certain behavior instead of trying to extinguish the behavior altogether. For IVP, an approach that emphasizes positive IVP practices among FEWs and health care providers including external cleaning for the physical and psychological benefits while discouraging internal cleaning without judgment, coercion, or discrimination. This may be an important way to promote positive messages about the desire to feel clean as well as address some of the sources of internalized and enacted stigma in their lives.

## Data Availability

The dataset comprised of focus group and interview transcripts is available upon request. Please contact the corresponding author: Dr. Carinne Brody, carinne.brody@gmail.com.

## Abbreviations

FEWs: Female entertainment workers
FGD: Focus group discussions
HIV: Human immunodeficiency virus
IDI: In-depth interviews
IVP: Intra-vaginal practices
STI: Sexually transmitted infections
